# CRISPR-based gene expression control for synthetic gene circuits

**DOI:** 10.1042/BST20200020

**Published:** 2020-09-23

**Authors:** Javier Santos-Moreno, Yolanda Schaerli

**Affiliations:** Department of Fundamental Microbiology, University of Lausanne, Biophore Building, 1015 Lausanne, Switzerland

**Keywords:** CRISPR, gene expression and regulation, synthetic biological circuits, synthetic biology

## Abstract

Synthetic gene circuits allow us to govern cell behavior in a programmable manner, which is central to almost any application aiming to harness engineered living cells for user-defined tasks. Transcription factors (TFs) constitute the ‘classic’ tool for synthetic circuit construction but some of their inherent constraints, such as insufficient modularity, orthogonality and programmability, limit progress in such forward-engineering endeavors. Here we review how CRISPR (clustered regularly interspaced short palindromic repeats) technology offers new and powerful possibilities for synthetic circuit design. CRISPR systems offer superior characteristics over TFs in many aspects relevant to a modular, predictable and standardized circuit design. Thus, the choice of CRISPR technology as a framework for synthetic circuit design constitutes a valid alternative to complement or replace TFs in synthetic circuits and promises the realization of more ambitious designs.

## Introduction

The human species embraces engineering endeavors in every aspect of life, and biology is no exception. Synthetic biology is a promising multidisciplinary field that uses concepts from engineering (such as abstraction, standardization and modularity) to design and create, rather than to only observe and perturb, biological systems. The interface between biology and engineering is successfully growing in areas as varied as metabolic engineering [1], biocomputing [2], cell-based therapeutics [3], biosensors [4], biomaterials [5], tissue engineering and artificial patterning [6,7], genetic re-coding [8], or synthetic/minimal life [9]. One of the fundamental goals of synthetic biology is to create gene circuits that allow for precise spatiotemporal control of gene expression [10]. In nature, gene circuits allow living organisms to respond to intra- or extracellular cues or conditions. Synthetic gene circuits provide researchers with similar programming capabilities and constitute key elements for the success of most engineering efforts in the areas of research listed above. Moreover, the construction and analysis of synthetic circuits that are simplified and controllable versions of natural ones revealed to be a powerful approach for understanding natural gene networks, which often are not amenable to study for complexity or essentiality reasons. Examples of successful efforts to construct synthetic circuits include logic gates [11], bistable switches [12,13], memory devices [14,15], signal-dependent and -independent patterning circuits [16–18], counters [19], light-pattern- and edge-detectors [20,21], intercellular communication circuits [22,23], or molecular clocks [24–26].

Most of the synthetic circuits developed so far employ protein regulators (i.e. transcription factors, TFs) to control gene expression. While protein-based regulators generally offer robust behaviors, they suffer from inherent constraints that limit their applicability from an engineering perspective, such as insufficient modularity, orthogonality and programmability. The use of CRISPR-based circuits promises to bypass many of those limitations. Here we provide an overview of the ever-expanding CRISPR technology for synthetic circuit construction, its possibilities and limitations, and the successful endeavors that have produced CRISPR-based synthetic circuits.

## CRISPR mechanism and applications

CRISPR refers to a defense mechanism that is employed by bacteria and archaea to inactivate exogenous nucleic acids, such as plasmids or phage genomes [27]. The basic mechanism of CRISPR immunity is simple. When an exogenous nucleic acid invades the cell, its identity is recorded in a particular cluster of the host genome (the CRISPR array) in the form of short stretches of the invader sequence. This cluster is transcribed and processed into short RNA molecules able to recognize invading sequences and guide the defensive response against them. Thus, during subsequent attacks, specific endonucleases associated to such ‘guide' RNAs will bind to and cleave the invading nucleic acid. The exogenous DNA (or RNA) is identified through a combination of two signals: a stretch (the ‘protospacer') with perfect complementarity to the 5′ region (the ‘spacer') of the guide RNA, and an adjacent short motif (named the protospacer adjacent motif, PAM) recognized by the endonuclease. Some CRISPR types actually utilize two RNA molecules (the CRISPR RNA, cRNA; and the trans-activating CRISPR RNA, tracrRNA) complexed to the nuclease, but chimeric single guide RNAs (sgRNAs) have been engineered for enhanced versatility and straightforward design. For simplicity, here we generically refer to guide RNA (gRNA) throughout the text.

The most immediate application of CRISPR is as highly programmable and specific molecular scissors [28]. By modifying the short (commonly 20 nt) spacer sequence of the gRNA, the system can be easily targeted to virtually any DNA sequence of interest. The *Streptococcus pyogenes* CRISPR associated protein 9 (Cas9, the most studied CRISPR nuclease) [29] has largely transformed the field of gene editing. Cas9-mediated cleavage enables gene editing through double-strand breaks (DSBs) and subsequent cellular repair [30], and even user-defined modifications when ‘donor’ template sequences are provided [31,32]. Moreover, nuclease-null (the catalytically ‘dead’ dCas9) or nickase (nCas9) variants of the CRISPR nucleases have also been employed, often fused to additional ‘effector’ domains, to perform precise (single-base) editing of genomic DNA without the need for donor templates or DSBs [33–35].

dCas9 and equivalents, alone or in combination with effector domains, have been extensively used for applications requiring cleavage-free specific DNA binding. These include gene expression control (dubbed CRISPRa and CRISPRi, for CRISPR activation and interference, respectively) [36,37], loss-of-function (via CRISPRi) or gain-of-function (using CRISPRa) screens [38,39], gene circuit construction [40,41], epigenome engineering [42–44], CRISPR-ChIP (chromatin immunoprecipitation) [45,46] and genome visualization [47,48]. Likewise, other CRISPR proteins with alternative functions offer attractive characteristics and have been harnessed for varied applications, such as polycistronic mRNA cleavage [49–51], molecular recording [52,53], post-transcriptional regulation [54], or highly sensitive and specific DNA and RNA detection – including detection of human viruses such as Dengue, Zika and SARS-CoV-2 coronavirus [55,56].

## Why CRISPR circuits

One of the applications where the use of CRISPR tools holds a vast potential is the construction of synthetic gene circuits. Since the publication of the first artificial gene circuits in the year 2000 [12,24], most synthetic circuits have made use of (natural, mutant or synthetic) TFs as a means to regulate gene expression. Generally, TFs offer important characteristics for gene circuit function such as a high dynamic range (i.e. difference between OFF and ON states) and frequently also an ultrasensitive behavior (i.e. a switch-like activation/repression), but they are far from ideal from an engineering point of view.

TFs are difficult to engineer: their specificity relies on protein-DNA binding, an interface that is immensely more difficult to predict, model and design than DNA-DNA or RNA-DNA binding. Despite notorious efforts to develop modular TFs – e.g. transcription activator-like effector (TALE) TFs [57] or zinc finger TFs [58] – their designability still remains limited [59,60]. Another problem relates to the available choice of TFs: because the number of well-characterized TFs is still rather modest, synthetic biologist often use TFs from the same superfamilies. However, this often results in a limited orthogonality, i.e TFs binding to each other's binding sites, albeit with lower affinity – this is particularly troublesome when building large circuits. Despite notable exceptions [20,61], large circuits are also likely to suffer from ‘incremental burden’: as the number of TFs increases, the host cell needs to cope with additional transcription and, more importantly, translation, the latter being a highly demanding process that can notoriously impact the host metabolism. Moreover, sequences encoding TFs are relatively long – for instance, the median length of proteins involved in transcription is 240 aa in bacteria and 444 aa in eukaryotes, which corresponds to 720 nt-long and 1332 nt-long genes, respectively [62]. Longer encoding sequences result in higher synthesis costs and more challenging cloning and delivery to the host cell.

In CRISPR circuits, the binding of the effector protein (nearly always dCas9 or a derivative) to the DNA is mediated by an associated RNA molecule, the gRNA, and this effectively circumvents many of the intrinsic limitations of TFs. CRISPR-mediated gene expression control is extremely programmable and easy to design – the modification of the 20 nt spacer sequence of the gRNA suffices to direct the activity of dCas9 to a new target. For the same reason, CRISPR orthogonality is virtually unlimited, with a theoretical upper limit of 4^20^ possible sequences of 20 nt. Although this number is practically unrealistic, because highly similar sequences are likely to exhibit some degree of crosstalk, the pool of potentially orthogonal gRNAs is way larger than the number of gRNAs that a researcher would possibly be able to use in a rationally-designed circuit. The ribonucleic nature of the gRNAs allows for straightforward and reliable *in silico* prediction of their structure and behavior in different environments, which facilitates the extension of the gRNAs with functional modules conferring new functions, such as ligand-binding RNA aptamers [63,64]. Besides, gRNA sequences are short (typically ∼100 nt for the most popular gRNA), which positively impacts design, synthesis economy, cloning procedures and delivery to the host cell.

Despite existing reports on (d)Cas9 toxicity [65–69], which has been proposed to be sequence-specific [70], the incremental burden of CRISPR circuits is generally low. Given a basal expression of dCas9, the extension of a circuit through the addition of extra regulatory interactions does not pose an insurmountable challenge on the host metabolism, since only transcription of the gRNAs (but not translation) is required [69]. Indeed, this low incremental burden facilitated in 2017 the construction of the largest synthetic circuit hitherto [71]. The production of the relatively large dCas9 might, in some instance, challenge the host metabolism, especially when high dCas9 levels are required or when (small) fitness variations introduce a high penalty – e.g in a poor medium or in a competition assay. Yet, with a steadily increasing number of available CRISPR systems, such considerations may soon become obsolete – for instance, a hypercompact CasΦ has been recently discovered that is about half the size of Cas9 [72]. Alternatively, novel resource-effective designs may help mitigate the potential load on the host metabolism, for example by engineering self-adjustable dCas9 expression systems [73].

## Gene expression control using CRISPR

**Figure 1.**
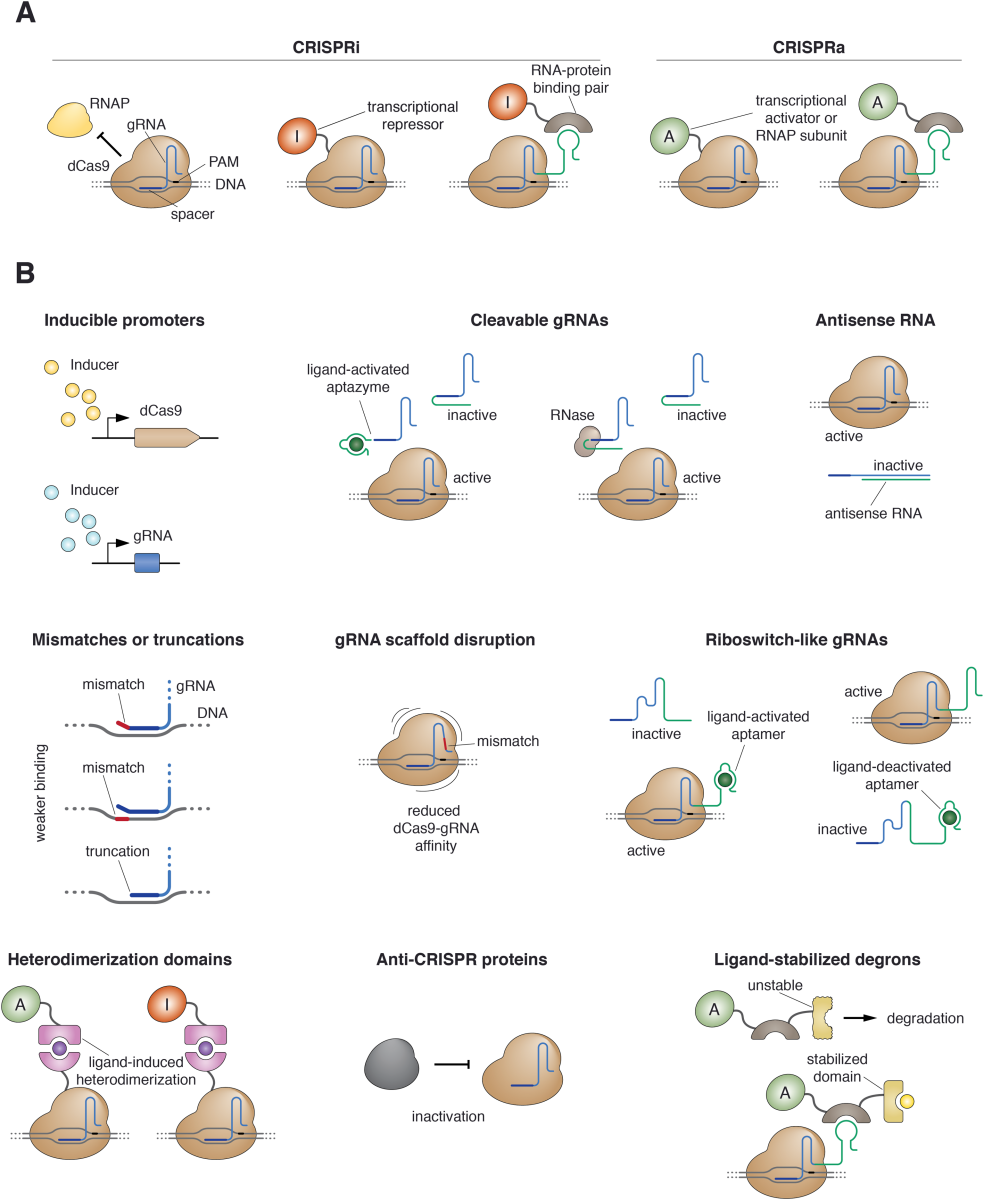
Gene expression control through CRISPRi and CRISPRa, and strategies to modulate their activity. (**A**) Mechanisms of CRISPRi/a-based gene expression control. The activity of ‘helper’ effector domains is required except for bacterial and yeast CRISPRi, where dCas9 alone can efficiently repress gene expression by interfering with transcription initiation or elongation. These effector domains can be either fused to dCas9 or recruited through an additional RNA-recognizing domain that binds to a specific extension of the gRNA. (**B**) Strategies to modulate CRISPRi/a activity. CRISPR gene control has proved a versatile platform that tolerates numerous engineering extensions. Due to its dual protein-RNA nature, the activity of the dCas9-gRNA complex can be modulated at the level of the protein (dCas9), at the level of the gRNA, or at the interface between the two. Note that for simplicity this panel depicts dCas9 alone, but many of these tuning strategies actually also work with dCas9 fused to effectors.

The general requirement for the use of CRISPR technology in gene circuits is the ability to regulate gene expression. The strategies for transcriptional activation and repression through CRISPR tools are known as CRISPRa (activation) and CRISPRi (interference), respectively (Table 1 and Figure 1A). In bacteria, the mechanism for CRISPRi is rather simple: repression can be achieved by directing a dead CRISPR nuclease (most commonly dCas9) to a transcriptional promoter or a downstream coding sequence (CDS) (Figure 1A) [37,74]. Promoter targeting is more effective because it blocks the binding of the RNA polymerase (RNAP) to the DNA, thereby preventing transcription initiation. Targeting the CDS works as a roadblock for transcriptional elongation, and its efficiency decreases with increasing distance between the binding site and the promoter. In yeast, dCas9 is an efficient transcriptional repressor both alone and in combination with the Mxi1 silencing domain [36,75]. In mammalian cells, dCas9 alone is not an efficient repressor [37], and needs to be fused to a repressive domain such as KRAB (Krüppel-associated box) (Figure 1A) [36,75].

**Table 1:** The CRISPR toolbox for gene expression control

Cas protein	Fusion	Function	Cellular context	Reference(s)
dCas9	-	CRISPRi	*E. coli* and other bacteria, yeast	[36,74,75,84]
dCas9	RNAP omega subunit	CRISPRa	*E. coli*	[74]
dCas9	MS2 aptamer (in gRNA) that recruits MCP-SoxS	CRISPRa	*E. coli*	[77,78]
dCas9	BoxB aptamer (in gRNA) that recruits λN22plus-PspFΔHTH	CRISPRa	*E. coli*	[79]
dCas9	AsiA	CRISPRa	*E. coli*	[76]
dCas9	KRAB	CRISPRi	Yeast, human cells	[36,75,85–87]
dCas9	Mxi1	CRISPRi	Yeast	[36]
dCas9	SRDX	CRISPRi	*Arabidopsis*	[88]
dCas9	VP64	CRISPRa	Yeast, human cells, *Arabidopsis*, tobacco	[36,75,80,88,89]
dCas9	p65	CRISPRa	Human cells	[36]
dCas9	SunTag-VP64	CRISPRa	Human cells	[81,85]
dCas9	VPR (VP64-p65-Rta)	CRISPRa	Yeast, fruit fly, mouse and human cells	[82]
dCas9	SAM: dCas9-VP64 & MS2 aptamer (in gRNA) that recruits MCP-p65-Hsf1	CRISPRa	Mouse and human cells	[39]
dCas9	TV (tandem TAL and VP64)	CRISPRa	*Arabidopsis*, rice	[90]
dCas9	VP64 & MS2 aptamer (in gRNA) that recruits MCP-VP64	CRISPRa	*Arabidopsis*, rice	[91]
dCas9	MS2, PP7 or Com aptamer (in gRNA) that recruits MCP, PCP or Com fused to VP64 or KRAB	CRISPRa/i	Yeast, human cells	[83]
dCas9	p300	Histone acetylation	Human cells	[44]
dCas9	HDAC3	Histone deacetylation	Mouse cells	[92]
dCas9	PRDM9	Histone methylation	Human cells	[93]
dCas9	DOT1L	Histone methylation	Human cells	[93]
dCas9	LSD1	Histone demethylation	Mouse cells	[94]
dCas9	DNMT3a	DNA methylation	Mouse and human cells	[42,95]
dCas9	TET1	DNA demethylation	Mouse cells	[95]
dCas9	TET1 & MS2 aptamer (in gRNA) that recruits MCP-TET1	DNA demethylation	Human cells	[43]
dCas12	-	CRISPRi	*E. coli*, human cells	[96–98]
dCas12	KRAB	CRISPRi	Human cells	[98]
dCas12	SRDX	CRISPRi	*Arabidopsis*	[99]
dCas12	VP64	CRISPRa	Human cells	[98]
dCas12	p65	CRISPRa	Human cells	[100]
dCas12	VPR (VP64-p65-Rta)	CRISPRa	Human cells	[98]
Cas13	-	RNA transcript knockdown	*E. coli*, rice and human cells	[101]

CRISPRa requires activator domains both in eukaryotes and prokaryotes (Table 1 and Figure 1A). In bacteria, dCas9 has been fused to the RNAP omega subunit [74] or to the phage activator AsiA [76]. Alternatively, the gRNA has been extended with protein-recruiting RNA scaffolds (Figure 1A), such as the MS2 hairpin that recruits a MCP (MS2 coat protein)-SoxS activator fusion protein [77,78], or the BoxB aptamers that bind to the λN22plus peptide fused to the PspF activation domain to promote gene expression from σ^54^ promoters [79]. Likewise, in eukaryotic systems the activator domain works as a direct dCas9 fusion (for example the VP64 domain, the tripartite VPR, or the repetitive SunTag) [75,80–82], indirectly as a fusion to an RNA-recognizing peptide (like the MS2-MCP pair) [83], or by combining these two approaches (e.g. the synergistic activation mediator, SAM) [39]. Table 1 summarizes the different CRISPR proteins and their fusions used for CRISPRi, CRISPRa and epigenome-modifying functions.

## Possibilities and limitations

A key feature of CRISPR gene control for building complex circuits is multiplexing, i.e. the ability to regulate multiple targets at the same time [102]. CRISPR-based regulation is intrinsically modular: the target is chosen by a gRNA, while the action on that target is mediated by the associated ‘effector’ protein (e.g. dCas9). Therefore, the same effector can be directed to multiple targets simply by using different gRNAs, which allows to simultaneously control tens of genes [103,104] and even to activate some genes while repressing others [83,105]. Of course, orthogonality is a prerequisite for multiplexing; the design of orthogonal gRNA spacers or the use of orthogonal CRISPR systems from different species [106] allows to easily avoid cross-reactivity.

One of the downsides of multiplexing is retroactivity, i.e. a decrease in performance of CRISPR-mediated regulation with increasing numbers of gRNAs due to competition for a common pool of dCas9 molecules [107–109]. The pool of dCas9 may be regulated to limit retroactivity: recently, optimization of dCas9 expression levels enabled efficient repression of up to 22 gRNA targets simultaneously [104]. This should be sufficient for most synthetic circuits, especially since often not all gRNA are expressed at the same time but rather regulate each other in a concerted manner [69,109].

When trying to express multiple gRNAs, instead of expressing them from separate promoters, strategies exist to combine them within a single transcriptional unit and to process the transcribed mRNA molecule into independent gRNAs. This simplifies the design, reduces the probability of unintended recombination due to repetitive promoter/terminator sequences, and allows for combined control of gRNA expression. Unintended recombination may also result from the repetition of gRNAs sharing the same nuclease-binding scaffold. To address this issue, a recent work developed non-repetitive gRNA scaffolds that enabled the simultaneous expression of more than 20 gRNAs [104]. In some natural CRISPR systems, the effector nuclease also works as a sequence-specific endoribonuclease (e.g. Cas12a), which enables the use of multi-gRNA constructs without additional RNA processing tools [103]. Some other CRISPR systems employ accessory proteins to cleave the multi-gRNA transcript – for instance Csy4 [110], which has been used as a gRNA-processing tool in (d)Cas9-based setups [40,50,51]. Alternatively, self-cleaving ribozymes have also been used to process multi-gRNA transcripts [40].

An important consideration when working with CRISPR is the target specificity. Unintended off-target activity has been extensively reported [111,112], and constitutes one of the main limitations of CRISPR technology, especially for applications where exquisite precision is needed, like gene editing for human therapeutics. The lack of specificity is more pernicious in eukaryotes because larger genomes increase the number of potential unintended binding sites. There is consequently a major interest in developing CRISPR tools with improved specificity, and numerous successful approaches have been reported [35,49,113–116]. Interestingly, CRISPRi and CRISPRa show reduced off-target levels compared to CRISPR-mediated DNA cleavage [36,39], possibly due to the requirement for binding in the proximity of a promoter in the former [117], which makes it less likely for an off-target binding to be deleterious. The off-target activity is likely a major contributor of (d)Cas9 toxicity, and approaches that increase binding specificity can concomitantly reduce toxicity [108].

Regardless of the RNA-guided nature, CRISPR systems do not display fast dynamics. In fact, CRISPR target search requires a constant DNA interrogation by (d)Cas9 in search for PAM sites, which are very abundant across the genome (e.g. the dCas9 PAM NGG is present every 8 bp on average in the *E. coli* genome), and then unwinds the adjacent DNA and assesses gRNA complementarity [118]. Studies in *E. coli* and *Lactococcus lactis* suggest that a single fluorescently labelled dCas9 molecule requires few hours (∼4–6 h) to find a single target within the cell, which means that dCas9 binds for ∼20–30 ms to each potential target [119,120]. For comparison, a single fluorescently labelled LacI dimer in *E. coli* needs less than 5 min to find a single target, and spends <5 ms (the detection limit) bound to each potential target [121]. In real-world applications such as synthetic circuits, the amount of intracellular dCas9 is well above one molecule, which effectively reduces the target search time to few minutes [119,120]. Once bound to its target, the dissociation rate of dCas9 is low, with DNA replication being the major driver of unbinding [119].

One of the prerequisites for CRISPR-based gene regulation to replace TFs in gene circuits is to achieve a sufficiently high dynamic range. While the dynamic range of TFs is generally high, that of CRISPRi and CRISPRa systems seems to depend to a broad extent on the particular implementation. With a steadily growing number of available CRISPR-based gene activation and repression strategies, the dynamic range is not a major limitation anymore for most CRISPR circuit applications in both prokaryotes and eukaryotes. Only CRISPRa in bacteria is lagging behind, possibly hampered by the limited number of gene expression activation tools in bacteria, but recent studies reported up to 1000-fold activation [74,76,77,79].

Another potential limitation of CRISPR circuits is the apparent lack of non-linearity [41,122,123]. In TFs, the cooperative activity of multimers or the presence of multiple binding sites commonly results in a non-linear, ultrasensitive behavior characterized by a sigmoidal dose-response with a steep output increase for a narrow input range. Since dCas9 works as a monomer and associates to a single binding site, its dose-response was thought to be linear, which would greatly limit the broad applicability of CRISPR tools for synthetic circuit construction [41,122,123]. Indeed, a lack of non-linearity poses major challenges for building important synthetic networks such as oscillating or multistable circuits, which require non-linear gene expression control to operate [124–127]. However, recent results demonstrate that it is actually possible to build CRISPR-based multistable and dynamic circuits such as a toggle switch and an oscillator (see ‘CRISPR-based synthetic circuits' section for details) [69,128,129].

## Modulating CRISPRa/i activity

A central aspect of any circuit design is how to precisely modulate gene expression. Many TFs can be externally regulated by simple actions such as adding a small molecule to the medium, changing the temperature or the pH, or exposing cells to a light of a given wavelength. Unfortunately, no such regulation of nuclease activity has been found in native CRISPR systems. The simplest way to regulate CRISPR activity is to express either the gRNAs, the effector, or both, from an inducible promoter (Figure 1B). Yet, more sophisticated approaches for conditional CRISPR regulation have been developed (Figure 1B). In riboswitch-like gRNAs, for example, an RNA aptamer appended to the gRNA sequence undergoes conformational changes upon ligand binding that disrupt or restore the native gRNA structure, thereby abolishing [130] or enabling [63,130] function. Similarly, strand displacement reactions allow for disruption or reconstitution of a functional gRNA only in the presence of a trigger RNA molecule [131–134]. The availability of the gRNA can also be modulated by incorporating protein- or oligonucleotide-controlled RNA-cleaving units [135] or a ligand-responsive self-cleaving catalytic RNA (aptazyme) into the gRNA (Figure 1B) [64]. Additionally, inducible heterodimerization domains allow for colocalization of regulatory domains and DNA-bound gRNA-dCas9 complexes only in the presence of the inducer [105].

The strengths of CRISPR activation and repression can also be modulated by partially disrupting the DNA-gRNA or the gRNA-effector interactions (Figure 1B). For instance, truncations of the 20 nt spacer or mismatches between the spacer and its target reduce the binding strength and the subsequent regulation efficiency in a reasonably predictable manner. Moreover, short 5′ truncations of the gRNAs have interesting (and somehow unexpected) effects: 17–18 nt spacers result in reduced off-target activity without losing on-target efficacy [113], and even shorter spacers (15 nt) prevent DNA cleavage but still allow DNA binding and transcriptional repression, even when using catalytically-active Cas nucleases [103]. The magnitude of the regulation can also be tuned down by introducing mismatches in the gRNA scaffold, which presumably decreases gRNA-dCas9 affinity [79]. Other strategies for CRISPR activity modulation include the use of ligand-stabilized degron domains [136], viral anti-CRISPR proteins [137], light-induced CRISPR proteins [138,139] and antisense RNA molecules that bind and sequester gRNAs (anti-gRNAs) (Figure 1B) [67].

## CRISPR-based synthetic circuits

By definition, a circuit implies connecting components together into a larger, higher-level design. In that sense, individual circuit components should ideally feature two important properties: the amenability to form layered regulatory cascades; and the capacity to integrate multiple inputs. The former implies that regulatory interactions can be connected to each other without excessive loss of signal. The latter is key for implementing multi-input logic gates and for building branched networks.

**Figure 2.**
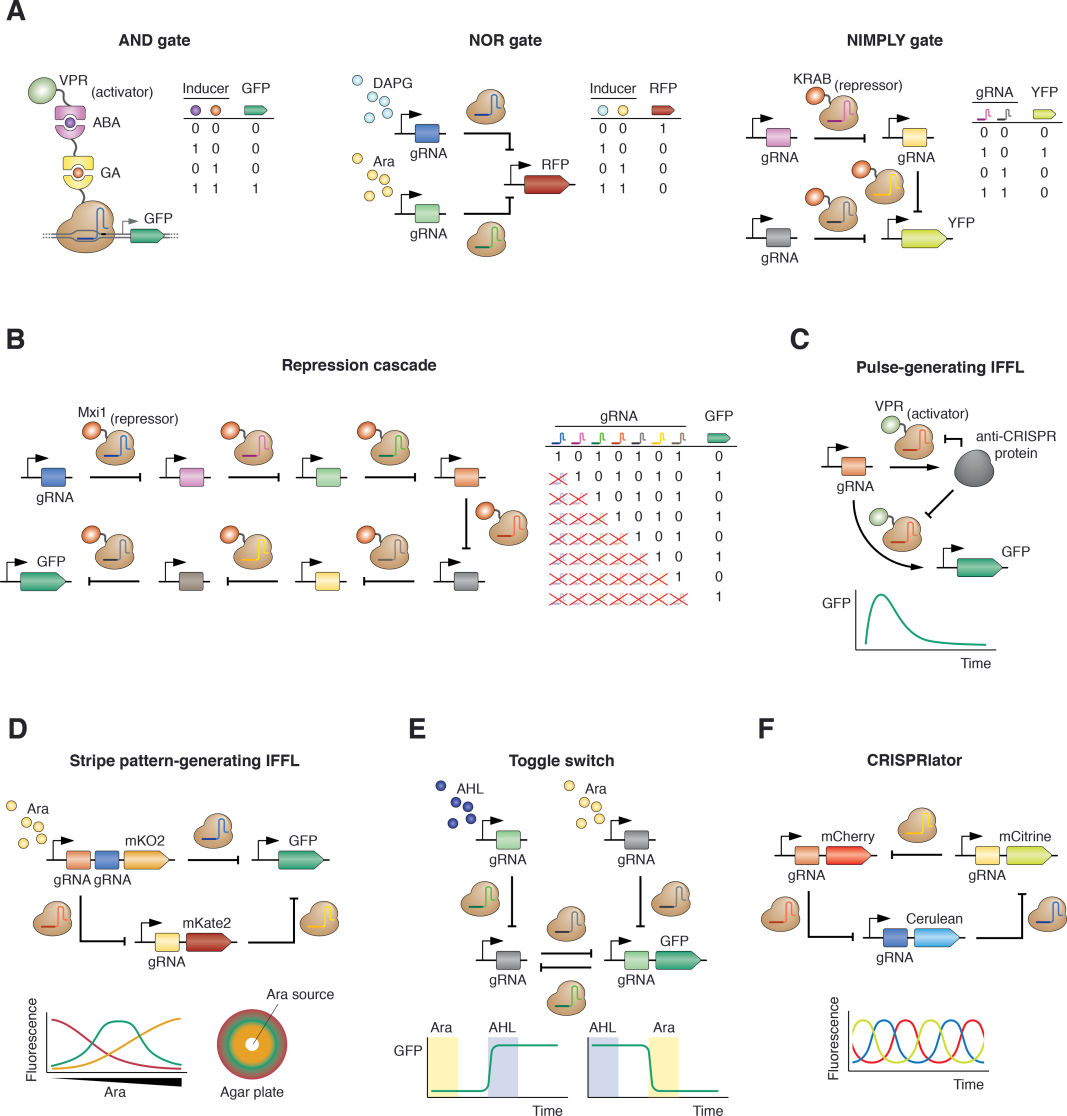
CRISPR-based synthetic gene circuits. (**A**) Examples of CRISPR-based logic gates. Left: AND gate based on ligand-induced heterodimerization domains implemented in human cells [105]. Only in the presence of the two ligands can the activator domain be recruited to the DNA-bound dCas9 and activate GFP expression. Middle: NOR gate constructed in *E. coli* using gRNAs expressed from inducible promoters [41]. Both gRNAs bind to and repress RFP, and thus only in the absence of both inducers can RFP be expressed. Right: NIMPLY gate in human cells mediated by KRAB-fused dCas9 [142]. YFP expression is enabled when one of the input gRNAs (but not the other) is present. (**B**) Seven-layer repression cascade built in yeast by using dCas9-Mxi1 [71]. Each new layer inverts the ‘sign’ (i.e. 1 or 0, high or low) of the downstream layers and of the final output. (**C**) Pulse-generating IFFL in human cells by leveraging dCas9-VPR inactivation by anti-CRISPR proteins [137]. The result is a temporal peak of gene expression. (**D**) Stripe-forming IFFL in *E. coli* [69]. In the presence of a gradient of arabinose (Ara, the input inducer), the dCas9-governed circuit forms a peak (stripe) of gene expression at intermediate Ara concentrations. Genetically identical bacteria carrying this circuit form a 3-color pattern in an agar plate when subjected to an Ara gradient. (**E**) dCas9-controlled toggle switch in *E. coli* [69]. The circuit can be toggled between the two stable states (green and non-green) through the addition of inducers to the medium: after inducing one of the states, the cells remain in that state even in the absence of any inducer, and the same is true for the opposite state. (**F**) The CRISPRlator, the first CRISPR oscillator [69]. A dCas9-mediated closed-ring repression topology generates periodic oscillations in *E. coli* cells.

A number of CRISPR circuits have been built so far in bacteria, yeast and mammalian cells (Figure 2), and many of these constitute experimental demonstrations of efficient layering and signal integration provided by CRISPR tools. Both CRISPRa and CRISPRi can be layered in series to form linear cascades. In CRISPRa cascades, layered activation results in signal transmission with no sign change [40,79]. On the other hand, layered CRISPRi provides sign inversions with every new layer added: a simple repression will convert a high signal into a low one, but two serial repressions (a ‘double-inverter’) will generate a high signal again, and so on [41,50,69,71,122,140–142]. So far, up to seven CRISPRi layers have been successfully connected into a repression cascade (Figure 2B) [71].

Signal integration is a necessary requirement for building most logic gates. Simple activation by CRISPRa and repression by CRISPRi constitute 1-input Buffer (passes the input unchanged to the output) and NOT gates, respectively, but gates with more complex functions generally require at least 2 inputs. CRISPR gene regulation has been employed by different groups to build Buffer, NOT, AND, OR, NAND, NOR, XOR, XNOR, and NIMPLY gates (Figure 2A) [41,63,71,105,131,142,143] – some even in non-model organisms [144]. Since CRISPR gene regulation is essentially a two-component system (gRNA + effector), AND and NOR gates can be easily constructed simply by devising designs in which the two components are produced separately and both are required for function, i.e. gRNA + activating effector = AND gate, while gRNA + repressing effector = NOR gate. Alternatively, more elaborated mechanisms can be envisioned (Figure 2A). As repression interactions are sufficient to construct all types of logic gates, CRISPRi suffices and has been used to build all types of logic gates [41,71,142]. Despite the apparent simplicity of logic gates, they constitute powerful cell-programming tools with great potential. For example, CRISPR AND gates have been employed to selectively identify human bladder cancer cells and enable cancer-specific antitumoral activity [143].

CRISPR regulation has also been harnessed for modulating host physiology and biosynthetic pathways. CRISPRa and CRISPRi have proved suitable for tuning or redirecting fluxes in the endogenous *E. coli* maltose utilization pathway [41], in the heterologous violacein pathway [79,145], and in endogenous cell proliferation and cell migration oncogenic signaling networks [63]; also for preventing metabolic burden [146], for controlling bacterial replication [147], or for triggering DNA looping [148] or cell filamentation [149]. Furthermore, CRISPR-based synthetic circuits have also been used to produce temporal or spatial behaviors. For instance, combining CRISPRa or CRISPRi with additional regulatory approaches (such as anti-CRISPR proteins or small transcription activating RNAs, STARs) has enabled the construction of pulse-generating incoherent feed-forward loops (IFFL) (Figure 2C) [137,150]. In response to a spatial concentration gradient of an inducer molecule IFFLs can also produce a spatial change in gene expression, the so-called stripe pattern or band-pass filter. Such a CRISPR-based IFFL has been built to govern the 3-domain patterning of a bacterial population (Figure 2D) [69]. The high orthogonality and low incremental burden of CRISPR circuits facilitates the co-existence of such stripe-forming IFFL with additional circuits within the same cellular environment without cross-interference, including a second stripe-forming IFFL and a double-inverter [69].

As already mentioned, a potential limitation of CRISPR circuit is the apparent lack of non-linearity [151]. Without non-linearity, circuits with relevant behaviors such as bistable (toggle) switches and oscillators cannot be achieved. The linear dose-response of CRISPR gene regulation has been both modelled [123] and experimentally observed [41], thereby supporting the aforementioned constraint. However, a recent study demonstrated the use of CRISPRi to construct circuits with behaviors requiring non-linearity, including a CRISPRi bistable toggle switch (Figure 2E) and a CRISPRi oscillator (Figure 2F), named CRISPRlator [69]. The CRISPRlator displayed robust inheritance of the oscillatory state across cell divisions, resulting in synchronous long-term oscillations of a cell-population in a microfluidic chamber.

Further confirmation of dynamic CRISPR circuits comes from a subsequent study showing an alternative dCas9 oscillator as well as a dCas12a-based version [128]. In this setup, the dCas12a-based circuit displayed more regular oscillations than the one relying on dCas9 [128]. Additionally, a new preprint explored a hybrid oscillator in which one of the three initial TFs of the repressilator is replaced by a dCas9-gRNA complex [129]. In this study, decoy binding sites for the dCas9-gRNA complex have been suggested to play a role in generating the required non-linearity in the absence of cooperativity. Interestingly, other successful designs lack such specifically designed decoy binding sites [69,128]. Instead, mathematical modeling suggests that the repeated weak binding of dCas9 to the numerous PAM sites during its target search could provide the non-linearity required for these circuits to work [69]. Nonetheless, the conditions that determine the linear [41] versus non-linear [69,128,129] behavior of CRISPR gene regulation remain yet to be uncovered.

## Final remarks

CRISPR gene regulation offers an easy design, a notable dynamic range, and a good compatibility with other transcriptional regulation approaches [40,67,150,152]. The exceptional orthogonality and the low incremental burden enable the construction of remarkably large circuits or combinations of them [69,71]. Despite these many advantages of CRISPR tools, relatively few synthetic CRISPR circuits have been built so far. We anticipate that recent substantial contributions such as novel CRISPRa/i approaches or circuits displaying non-linear behaviors will boost the use of CRISPR for synthetic circuit construction in the following years.

## Perspectives

- Synthetic gene circuits allow us to program cells in a reliable and user-defined manner for a myriad of applications ranging from engineered cell-based therapeutics to ‘smart’ crops or sustainable bioproduction. CRISPR-based gene expression control holds great potential over ‘classical’ transcription factor (TF)-based designs for synthetic circuit construction due to favorable characteristics such as high programmability, modularity and orthogonality.
- Relatively few CRISPR-based gene circuits have been built so far, and their full potential has not yet been unleashed. Present CRISPR circuits include logic gates, cascades, bistable switches and temporal and spatial pattern generators, showing that TF-equivalent (or even improved) behaviors are possible.
- Recent advances such as improved and modulable CRISPR gene regulation or CRISPR circuits capable of non-linear behaviors have provided the field with new impulses. We anticipate that CRISPR circuits will enable the construction of complex and large synthetic circuits that would not be achievable with TF-based designs.

## Abbreviations

aa: amino acid
ABA: abscisic acid
AHL: *N*-acyl homoserine lactone
Ara: arabinose
AsiA: 10 kDa anti-sigma factor
Cas9: CRISPR associated protein 9
CDS: coding sequence
ChIP: chromatin immunoprecipitation
CRISPR: clustered regularly interspaced short palindromic repeats
CRISPRa: CRISPR activation
CRISPRi: CRISPR interference
CRISPRlator: CRISPR oscillator
cRNA: CRISPR RNA
Csy4: CRISPR-associated endonuclease Cas6/Csy4
DAPG: 2,4-Diacetylphloroglucinol
dCas9: catalytically dead Cas9
DNMT3a: DNA (cytosine-5)-methyltransferase 3A
DOT1L: DOT1 like histone lysine methyltransferase
DSB: double-strand break
GA: gibberellin
GFP: green fluorescent protein
gRNA: guide RNA
h: hour
HDAC3: histone deacetylase 3
IFFL: incoherent feed-forward loop
KRAB: Krüppel-associated box
LacI: *lac* repressor
LSD1: lysine-specific histone demethylase 1
MCP: MS2 coat protein
min: minute
mKO2: mKusabira-Orange 2
ms: millisecond
Mxi1: MAX interacting protein 1
nCas9: nickase Cas9
nt: nucleotide
PAM: protospacer adjacent motif
PRDM9: PR/SET domain 9
PspF: Psp operon transcriptional activator
RFP: red fluorescent protein
RNAP: RNA polymerase
RNase: ribonuclease
SAM: synergistic activation mediator
SARS-CoV-2: severe acute respiratory syndrome coronavirus 2
sgRNA: single guide RNA
SRDX: EAR repression domain
STAR: small transcription activating RNA
TAL: TALE transcription activation domain
TALE: transcription activator-like effector
TET1: Tet methylcytosine dioxygenase 1
TF: transcription factor
tracrRNA: trans-activating CRISPR RNA
VP64: four tandem copies of herpes simplex viral protein 16
VPR: VP64-p65-Rta tripartite activator
YFP: yellow fluorescent protein
